# Rapid impact assessments of COVID-19 control measures against the Delta variant and short-term projections of new confirmed cases in Vietnam

**DOI:** 10.7189/jogh.11.03118

**Published:** 2021-11-04

**Authors:** The-Phuong Nguyen, Zoie SY Wong, Lin Wang, Truc Thai Thanh, Huy Van Nguyen, Stuart Gilmour

**Affiliations:** 1 Graduate School of Public Health, St. Luke's International University, Tokyo, Japan; 2 Division of Surveillance and Policy Evaluation, Institute for Cancer Control, National Cancer Center, Tokyo, Japan; 3 Department of Genetics, University of Cambridge, Cambridge, UK; 4 University of Medicine and Pharmacy at Ho Chi Minh City, Ho Chi Minh City, Vietnam; 5 Health Innovation and Transformation Centre, Federation University, Victoria, Australia

## PREVIOUS SUCCESS OF ZERO-NEW-CASE-APPROACH IN VIETNAM

As of 2020, the cumulative number of COVID-19 cases recorded in Vietnam was less than 1500, proving the success of COVID-19 control in Vietnam [1]. Vietnam has been recognized as one of the few countries that successfully controlled COVID-19 in 2020 [2]. Several recent articles have summarised a set of lessons learned, the so-called “Zero-new-case-approach”. These included (i) a rapid and coordinated public health response with a decentralized health care system [3]; (ii) massive quarantine and targeted lockdown; (iii) third-degree contact tracing; (iv) centralized patient management; (v) early school closures and robust border controls; (vi) mask policies and 5K message (5K refers to use face masks in public places, disinfect regularly, keep distance, stop gathering, and make health declaration); and (vii) innovative mass testing strategies in the resource-constraint system (sample pooling strategy of PCR test with 2-7 swaps) [4], These “Zero-new-case-approach” strategies all focused on the non-pharmaceutical aspect of disease control. They aimed to maintain zero community transmission by establishing a comprehensive public surveillance system and enacted drastic measures with the support of the police and military forces.

## RECENT CHALLENCES FROM NEW DELTA VARIANT

The recent fourth wave of COVID-19 outbreak in Vietnam started on Apr 27, 2021. This largest outbreak was primarily centred in Ho Chi Minh City (HCMC). All the new cases identified since May 19, 2021, originated from the new B.1.617.2 (Delta) variant of SARS-CoV-2 [5]. The Delta strain was estimated as 40%-60% more contagious than the other highly contagious Alpha variant, which was already 60% more infectious than the original variant [6]. Although the “Zero-new-case-approach” strategies have demonstrated success in containing the three earlier waves of COVID-19 in Vietnam that occurred in 2020, there are concerns about whether these measures are sufficient in controlling the Delta variant in Vietnam. Therefore, a rapid impact assessment of the effectiveness of currently applied strategies is urgently needed. This brief report estimates the time-varying reproduction number to evaluate the effectiveness of the control policies and projects a realistic future outbreak based on the latest epidemiological parameters of the Delta variant and the confirmed case data in the HCMC, Vietnam. The findings of this study provide critical advice for emergency responders and policymakers in controlling the current COVID-19 outbreak in Vietnam.

**Figure:**
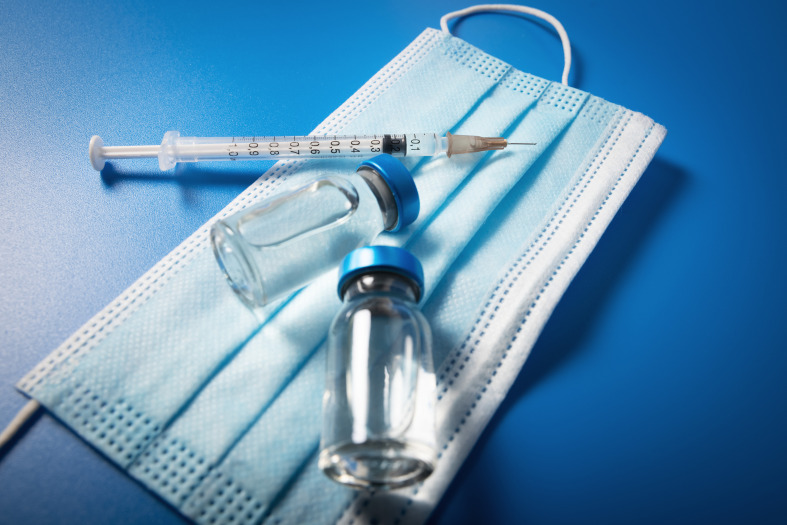
Photo: By ronstik via Pixabay.

## APPLICATION OF MATHEMATICAL MODELS IN RAPID IMPACT ASSESSMENT

We analyzed the time series laboratory-confirmed cases of the fourth wave of COVID-19 in Vietnam from the Ministry of Health Vietnam [1]. First, we examined the national epidemic curve stratified by region and calculated the 7-day moving average. Then, based on the estimated serial interval with Gamma distribution, we estimated the time-varying reproduction number (R) to understand changes in transmissibility over time in HCMC [7]. R was defined as the average number of secondary cases of disease caused by a single infected individual over the infectious period. The number of new cases will continue to increase when R>1, while there will be a decline in the number of new cases when R≤1. Thus, estimating R provides critical information on how the transmission rates fluctuated over time and how effective the recently implemented public health measures were. The formula is as shown below.



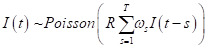


Where *I(t)* is the incidence onset at the time t, R is the time-varying reproduction number, and *ω* defines the serial interval distribution with an assumption of Gamma distribution of scale and shape parameters as 5.03 and 0.46 respectively [7]. Furthermore, using a stochastic branching process model with Poisson offspring distribution, we predicted future confirmed cases in HCMC in short-term time windows of 30 and 60 days [8]. Finally, we generated 10 000 simulations of joint posterior distributions from the initial conditions and R to estimate R's uncertainty and stochastic variability of the transmission process.

## INTERPRETATION OF EPIDEMIC CURVE, TIME-VARYING REPRODUCTION NUMBER AND STOCHASTIC BRANCHING PROCESS MODEL

**Figure 1:**
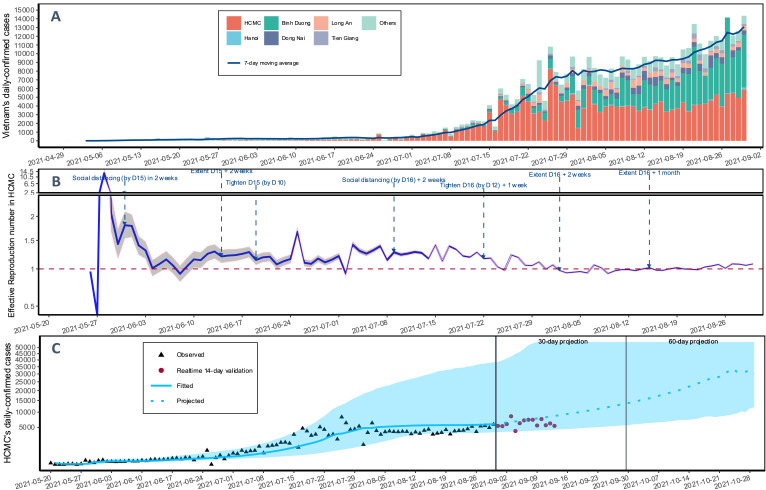
The fourth wave of COVID-19 in Vietnam. **Panel A.** Epidemic curve by provinces. **Panel B.** Time-varying reproduction number (**R**) and critical public health measures in HCMC. The red dotted horizontal line expresses the threshold of 1. When Re <1, the epidemic is under control. **Panel C.** Projections of daily new confirmed cases in HCMC in 30 and 60-day timeframe. HCMC – Ho Chi Minh city, D15 – Directive 15, D16 – Directive 16, D12 – Directive 12.

**Figure 1**, Panel A shows the epidemic curve of the fourth wave of COVID-19 in Vietnam from the first case found from April 27 to August 30, 2021, stratified by provinces. Since the first cases were identified on May 19, 2021, HCMC became the epicentre of the COVID-19 outbreak in Vietnam. Despite 4-month efforts, the Delta strain outbreak situation in Vietnam escalated. The daily number of confirmed cases in HCMC and the entire country was reported as 5889 and 14 352, respectively, on August 30, 2021, accounting for the total cumulative cases of 213 798 and 445 543 (**Figure 1**, Panel A).

As shown in **Figure 1**, Panel B, the Vietnamese government applied various non-pharmaceutical public health measures with an increased level of strictness: Directive 15 (May 31 to June 29), then raised by adding Directive 10 (June 19), and subsequently, Directive 16 (July 9 to September 15) with the tighten of Directive 12 (July 22). In general, all these directives contained four non-pharmaceutical aspects, including restricting large gatherings, social distancing, business and services, and transportation activities. Directive 16 is the strictest policy, which requires all people to stay at home, restricting gatherings to less than three people, ceasing all business activities and transportation services, and only allowing essential services to remain open. **Figure 1**, Panel B also presents the estimated reproduction number (R) in HCMC over the study period (May 19 to August 22), which has been chiefly (69 days out of 104 days; 70%) above the threshold level of 1. Although the estimated R seems to reduce overtime of implementing public health measures, the current R was still above one with 1.08 (95% CI = 1.07-1.09) as of today (August 30, 2021).

**Figure 1**, Panel C presents the stochastic branching process model estimates based on the observed case data from May 19 to August 30, 2021. The projections of future confirmed cases in HCMC in the next 30 days (September 29) and 60 days (October 29) were 13 395 (95% CI = 5554-152 780) and 32 042 (95% CI = 12 346-484 600), respectively. These projections were made on the basis that no additional public health measures are implemented and assuming the current trend continued. As shown in **Figure 1****,** Panel C, our model demonstrated a good fit with the observed training data (from May 19 to August 30, 2021), indicating that if no additional effective measures are put in place in short order, the future outbreak would likely follow our projected trajectory.

## IMPORTANCE OF CO-IMPLEMENTING MULTIFACETED PHARMACEUTICAL AND NON-PHARMACEUTICAL INTERVENTION STRATEGIES

Although these non-pharmaceutical interventions successfully controlled previous waves of outbreaks in 2020, the situation seems not to be appliable to this current Delta outbreak. We argue that the new and contagious Delta variant of SARS-CoV-2, which has already been spreading to 124 countries globally, plays a critical role in expanding the outbreak size. The increased infectivity poses enormous challenges in implementing the third-degree contact tracing and concentrated quarantine in HCMC as a more significant number of close contacts would need to be isolated. Furthermore, with limited human resources in epidemiology, contact tracing can be overloaded, and further burden the health system. Thus, a careful rethink is necessary to ensure a robust and sustainable health system in the long run. The current public health measures will need to be scaled up, become more agile and adapt to this super infectious variant.

As a low-to-middle-income country, Vietnam faces insufficient vaccine supply and ineffective deployment in the global pandemic [9]. As of August 30, only 20 million people were inoculated with at least one dose, accounting for only 20% population [10]. The vaccination coverage in Vietnam is much lower than the global average of 39%, the Asian average of 44%, and is amongst the lowest in South Asian countries [11]. As a resource-limited country with urgent needs, the Vietnam government should pay attention to increasing vaccine access through negotiations with worldwide vaccine initiatives, such as COVAX, the nearby closely linked countries, and vaccine manufacturers. Additionally, the government could consider applying fractionation of COVID-19 vaccine doses as a potential mass-vaccination strategy, especially when vaccines are in short supply globally [12]. There are some concerns regarding the reduced effectiveness of vaccines against the Delta Variant and potential resistance towards other strains such as the Lamda variant [13]. Thus the Vietnamese government needs to ramp up the vaccination program while maintaining other non-pharmaceutical measures, including social distancing and border controls.

Furthermore, it is imperative to decrease pandemic fatigue among people in Vietnam (pandemic fatigue is defined as the natural demotivation to follow recommended protective behaviours, precautions and restrictions relating to a pandemic due to its effect on prolonged public health and economic crisis) [14]. The impact of the previously effective “Zero-new-case-approach” may be discounted because of reducing people's adherence to public health measures and restrictions. The WHO has recognized pandemic fatigue as a global threat and recommended governments seek to reinvigorate previous approaches with four key strategies of understanding people, engaging people as part of the solution, allowing people to live their lives, but reducing risks acknowledging and addressing the hardship people are experiencing [14]. Thus, an appropriate approach to manage the COVID-19 transition is needed to prepare for upcoming outbreaks and implementing urgent public health measures [15].

This viewpoint provided the first impact assessments of current public health measures and short-term projection of future confirmed cases for the government and policymakers to devise strategies to adapt to the challenging situation posed by the Delta strain outbreak in Vietnam. However, some limitations need to be mentioned. First, we used the strict assumptions of closed population and homogeneity among demographic groups, which may be straightforward but suitable for our rapid assessment at this early stage. Still, it warrants further comprehensive research with the more complicated models that integrating structuring population. Further, our short-term projections of new confirmed cases were estimated based on the current trends with the assumption of unchanged future policy. Thus, although they cannot reflect the improvements in public health measures and policies implemented after the study period, they still provided a business-as-usual scenario in the next 30 and 60 days. Finally, the realtime 14-day validation (observed data in 14 days after the study period) showed that 100% of future data was in our uncertainty range, and 65% (9 out of 14) was close to our point estimations. Hence, although the adopted analysis required further sensitivity analysis and cautioned interpretations, our viewpoint provided initial evidence to support the Vietnamese government and policymakers in tailoring future public health measures to control COVID-19.

## CONCLUSION

In conclusion, our analysis suggests that the existing public health measures were effective but insufficient to control the current COVID-19 outbreak caused by the Delta variant in Vietnam. The causes may be attributed to the combined reasons of high transmissibility of the Delta variant, pandemic fatigue among Vietnamese people, and insufficient vaccine supply and deployment. Therefore, we urge the government and policymakers to pay attention to multifaceted pharmaceutical and non-pharmaceutical intervention strategies that include innovating the vaccination program under demand constraints, reinvigorating control measures and continuing non-pharmaceutical measures.
